# Supplementary material for: Memristor-based adaptive analog-to-digital conversion for efficient and accurate compute-in-memory
Source: Nat Commun. 2025 Nov 6;16:9749. doi: 10.1038/s41467-025-65233-w (PMC12592462; doi:10.1038/s41467-025-65233-w)
Supplement: Supplementary file 1 — Supplementary Information [file 41467_2025_65233_MOESM1_ESM.pdf]

Supplementary Information

Memristor-based adaptive analog-to-digital conversion for efficient and accurate compute-in-memory

Haiqiao Hong[1], Zhiyuan Du[1], Mingrui Jiang[1], Ruibin Mao[1], Yuan Ren[1], Fuyi Li[2], Wei Mao[2], Muyuan Peng[1], Wei Zhang[3], Zhengwu Liu[1,*], Can Li[1,*], Ngai Wong[1,*]

[1]Department of Electrical and Electronic Engineering, The University of Hong Kong, Hong Kong, China.

[2]Hangzhou Institute of Technology, Xidian University, Hangzhou, China.

[3]Department of Electronic and Computer Engineering, The Hong Kong University of Science and Technology, Hong Kong, China.

[*]Email: zwliu@eee.hku.hk (Z.L.); canl@hku.hk (C.L.); nwong@eee.hku.hk (N.W.).

Supplementary Note 1 Mathematical framework for Q-cell implementation in n-bit memristor-based ADC

The Q-cell architecture presented in the main text enables efficient voltage quantization through programmable memristor conductance. This section establishes the mathematical foundation for determining the required number of Q-cells in an n-bit ADC implementation, supporting the practical demonstration shown in **Figs. 2c-d** of the main text.

1.1 Q-cell count derivation

For an n-bit ADC implementation, the number of required Q-cells can be derived as follows:

Total quantization boundaries required: $2^n - 1$.

Each Q-cell maps two boundaries (lower and upper thresholds), except the first Q-cell which maps one boundary.

Total boundaries mapped by m Q-cells: $1 + 2(m - 1)$.

Setting this equal to the required boundaries:

$$1 + 2(m - 1) = 2^n - 1 \quad (1)$$

Solving for m yields:

$$\begin{gathered} 1 + 2(m - 1) = 2^n - 1 \\ 2m - 1 = 2^n - 1 \\ m = 2^{n-1} \end{gathered} \quad (2)$$

1.2 Boundary mapping configuration

The boundary index mapping follows this configuration:

Q-cell 1: $[,2^{n-1}]$.

Q-cell i $(1 < i \leq 2^{n-1})$: $[i - 1, 2^{n-1} + i - 1]$.

This configuration, as implemented in the 3-bit uniform ADC example (**Figs. 2c-d** of the main text), shows how 2^{n-1} Q-cells can effectively map all $2^n - 1$ quantization boundaries. The overlapping boundary ranges, enabled by the dual-threshold programmability of each Q-cell, provide the foundation for both uniform and adaptive quantization schemes.

Supplementary Note 2 Q-cell output decoding

The outputs of the Q-cells are processed by a thermometer-to-binary decoder, which converts the 4-bit thermometer-coded Q-cell outputs into 3-bit binary ADC codes. The decoding logic is defined by the following truth table **Supplementary Table 1**. For example: When the Q-cell output is 1110, the decoder produces the binary output 110. When the Q-cell output is 0111, the decoder produces the binary output 000.

Supplementary Note 3 Robustness analysis against reference voltage variations

To evaluate the robustness of the proposed ADC against practical voltage instabilities, we performed a comprehensive parametric analysis. The study focused on the impact of two primary non-idealities on the ADC's linearity metrics (INL and DNL): dynamic power supply noise and static DC drift, applied to the reference voltages V_H and V_L. The simulation was structured across a three-dimensional parameter space under experimental device variation, with results plotted in **Supplementary Fig. 5**. To ensure statistical significance, each parameter point was evaluated through 10 Monte Carlo simulation runs, with 1% random variation applied to memristor conductance.

3.1 Impact of dynamic power noise

Our analysis evaluates the ADC's intrinsic robustness to high-frequency dynamic power noise, which represents random fluctuations that cannot be compensated for by recalibration. The plots in first row of **Supplementary Fig. 5** (Voltage Drift = 0.0 mV) specifically illustrate this resilience.

For the 5-bit ADC under zero DC drift, the architecture maintains excellent linearity even under significant noise. Increasing noise from 0.05 mV to 10 mV results in only minor degradation of linearity, with the RMS of maximum DNL increasing from 0.423 LSB to 0.485 LSB, and INL increasing from 0.306 LSB to 0.344 LSB. Even at a high noise level of 20 mV, the ADC maintains robust performance, with DNL and INL remaining at 0.521 LSB and 0.464 LSB, respectively. This demonstrates the architecture's inherent stability in noisy mixed-signal environments, which is a crucial characteristic for reliable operation in practical CIM systems.

3.2 Impact of static DC drift

To quantify the impact of static DC drift, we analyzed ADC performance under two noise conditions representing low (1 mV) and moderate (10 mV) system environments. When the static DC offset increases from 0 mV to 50 mV, the results show different sensitivity levels depending on the background noise.

In the low-noise environment (1 mV), performance degradation is modest: maximum DNL increases from 0.565 to 0.604 LSB, while maximum INL rises from 0.375 to 0.417 LSB. However, under moderate noise conditions (10 mV), the impact becomes more pronounced, with DNL increasing from 0.485 to 0.731 LSB and INL from 0.344 to 0.523 LSB.

However, this highlights our design's core advantage: unlike conventional ADCs requiring complex peripheral compensation, our architecture enables in-situ recalibration by updating the conductance-boundary map. This directly adjusts quantization thresholds to compensate for drifted VH/VL levels without additional hardware overhead.

Supplementary Note 4 Synergy with array-level compensation techniques for non-idealities

The performance of a CIM system is influenced by non-idealities at multiple stages of the signal path. While our work introduces a novel ADC to address the quantization bottleneck, we acknowledge that the accuracy of the analog VMM result, prior to quantization, is critically impacted by factors within the crossbar array, such as device-to-device variations and input-dependent IR drop on access lines. Our proposed ADC is designed as a modular and independent component, making it complementary to, rather than mutually exclusive with, state-of-the-art techniques developed to mitigate these array-level challenges.

This modularity enables a synergistic system design. For example, the work by Roy et al.[1] proposes a system-level statistical error compensation (SEC) algorithm. This technique learns and applies input-scaling factors to pre-compensate for the non-linearity caused by bitline and source-line parasitic resistances (IR drop). The SEC method corrects the analog signal before it is aggregated, ensuring the integrity of the VMM operation itself. Our adaptive ADC can then be employed to efficiently and accurately quantize this compensated analog signal. The two approaches work in tandem, with SEC addressing array-level signal integrity and our ADC addressing the subsequent quantization efficiency.

Other works have focused on different aspects of the readout chain. Khaddam-Aljameh et al.[2] introduced a linearized current-controlled oscillator (CCO)-based ADC that employs a feed-forward compensation technique to counteract its own transfer curve non-linearity and read voltage variations seen by the ADC. Hsu et al.[3] developed a current-voltage-hybrid (CVH) readout scheme to enhance the signal margin and manage large bitline currents during accumulation. These solutions address different, specific challenges in the signal path. Our memristor-based adaptive ADC offers a unique and highly efficient solution for the quantization stage that could be integrated into a system employing these or other advanced readout circuits.

In summary, a robust, high-performance CIM system will likely incorporate a suite of solutions targeting different non-idealities. Our work provides a critical component for this system, a highly efficient, adaptive quantization engine, that is fully compatible with orthogonal techniques designed to improve the fidelity of the analog computation within the crossbar array.

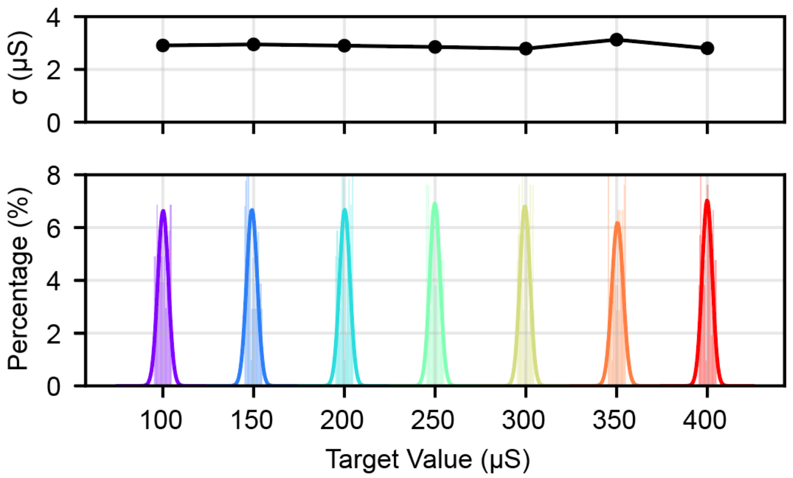

Supplementary Fig. 1. Program cycle-to-cycle (C2C) variation of a representative memristor device. The device was programmed to seven distinct target states 100 times each. The low standard deviation (SD: 2.90 µS, <1%) demonstrates high single-device repeatability.

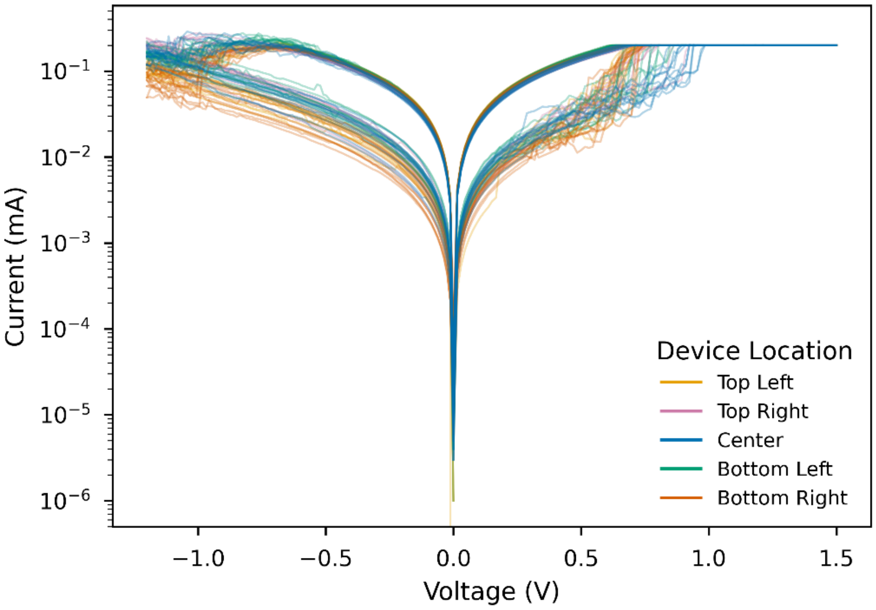

Supplementary Fig. 2. I-V switching characteristics of memristor devices from the 8×8 array. Ten consecutive voltage sweep cycles are shown for five devices at different array locations, demonstrating consistent bipolar switching behavior and uniform performance across the fabricated array.

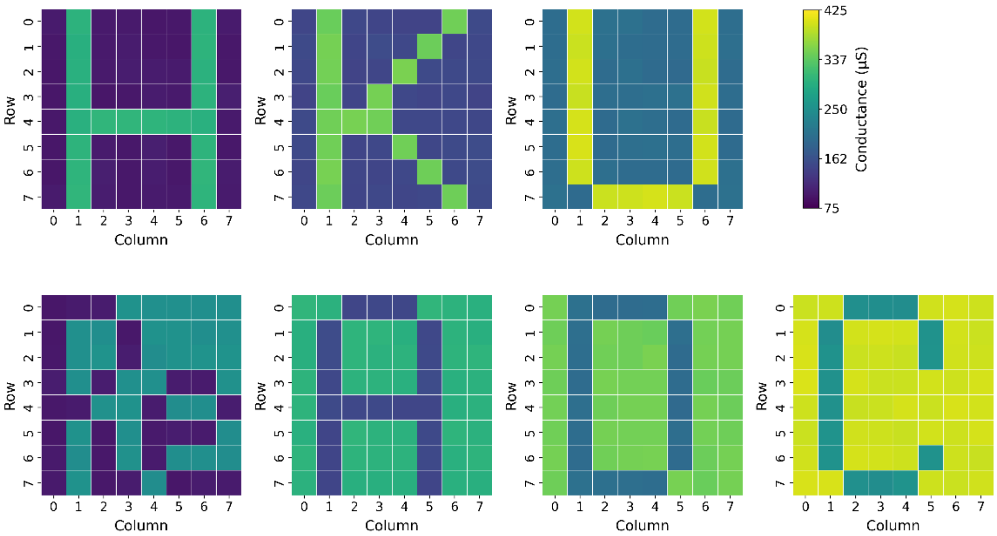

Supplementary Fig. 3. Demonstration of multi-state conductance programming on the 8×8 memristor array. Seven different letter patterns (‘H’, ‘K’, ‘U’, ‘Re’, ‘A’, ‘D’, ‘C’) were successfully programmed across the array with the color map representing measured conductance values. The clear formation of all patterns confirms high device yield and precise conductance state control across individual devices.

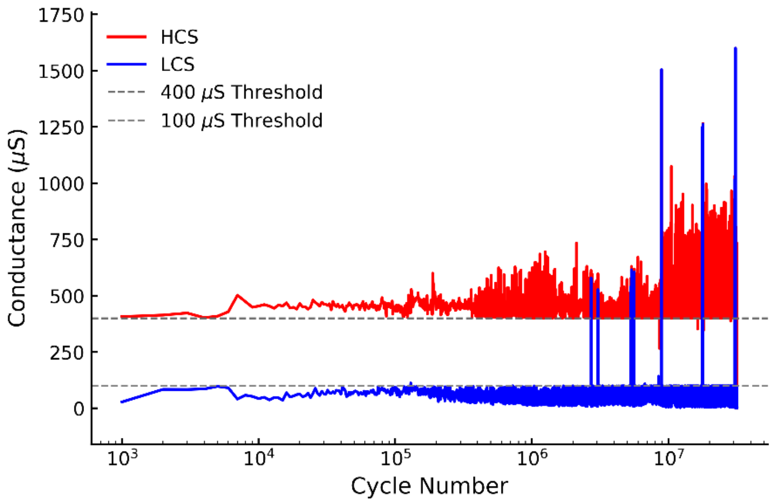

Supplementary Fig. 4. Experimental endurance characterization of the Pt/TaO_x/Ta memristor device. The device was subjected to repeated SET (target > 400 µS) and RESET (target < 100 µS) cycles. The high-conductance state (HCS, red) and low-conductance state (LCS, blue) are plotted against the cycle number on a logarithmic scale. The dashed lines indicate the operational conductance window required for the ADC application. The device demonstrates robust endurance, successfully completing over 3×10^7 cycles while maintaining a clear and stable distinction between the HCS and LCS states within the required operational window (Although some ultrahigh conductance states were observed during the final period, it can always recover to LCS after several reset trials), confirming its reliability for the intended application.

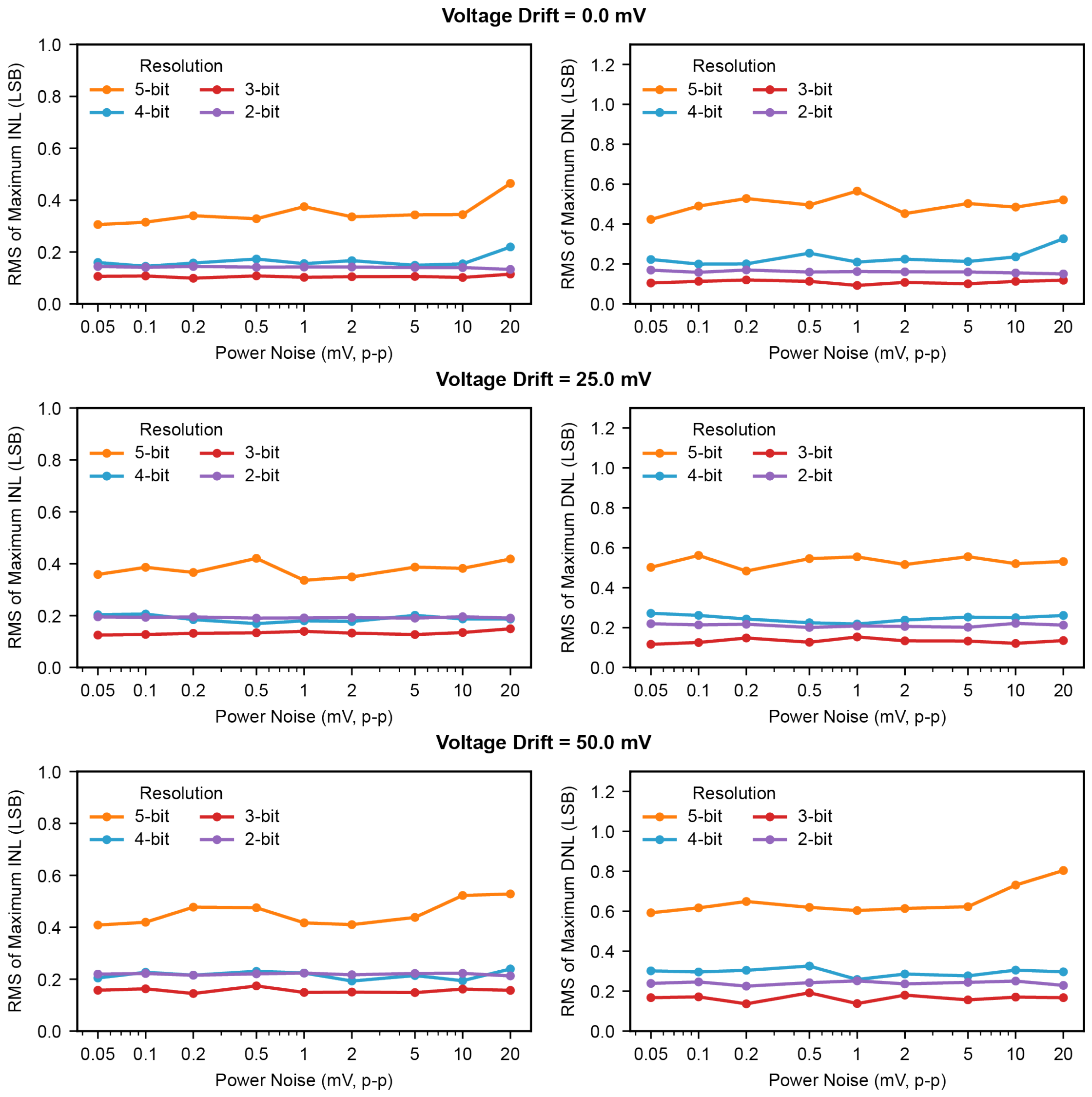

Supplementary Fig. 5. Simulated ADC linearity robustness against reference voltage variations. The plots show the RMS of the maximum INL (left column) and DNL (right column) for ADC resolutions from 2-bit to 5-bit. The analysis evaluates the impact of dynamic power noise (x-axis, ranging from 0.05 to 20 mV p-p) under three distinct static DC drift conditions (0, 25, and 50 mV) on the reference voltages V_H and V_L. Each data point represents the RMS of the maximum values gathered from 10 Monte Carlo simulation runs, ensuring a robust assessment of performance under a wide range of realistic operating conditions.

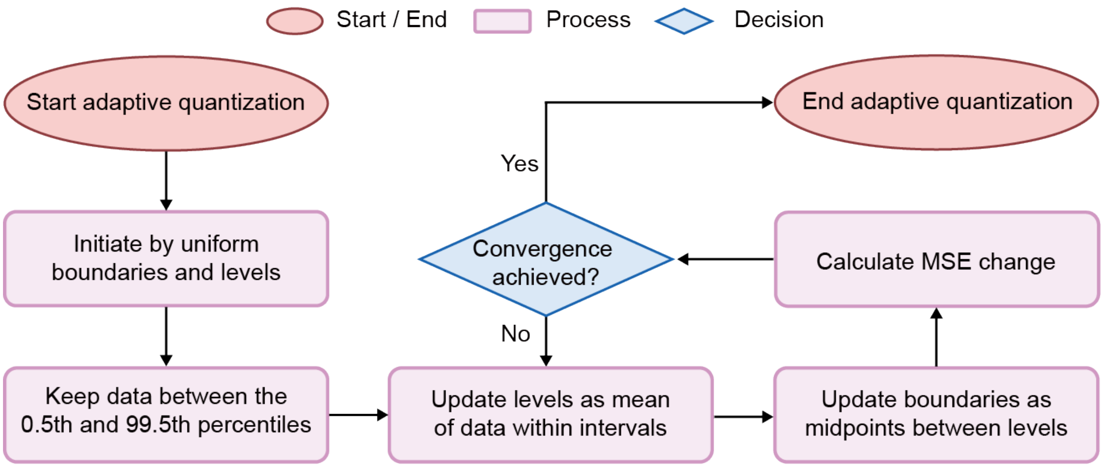

Supplementary Fig. 6. Flowchart of the memristor-based adaptive quantization process. This process optimizes quantization levels and boundaries to improve the efficiency and accuracy of CIM quantization operations. The algorithm iteratively adjusts quantization parameters until convergence is achieved, as indicated by the minimal change in MSE. The optimization continues until the change in MSE between iterations falls below the threshold. This approach fine-tunes the Lloyd-Max quantization scheme to improve performance on CIM array outputs, enhancing both the efficiency and accuracy of the analog-to-digital conversion process.

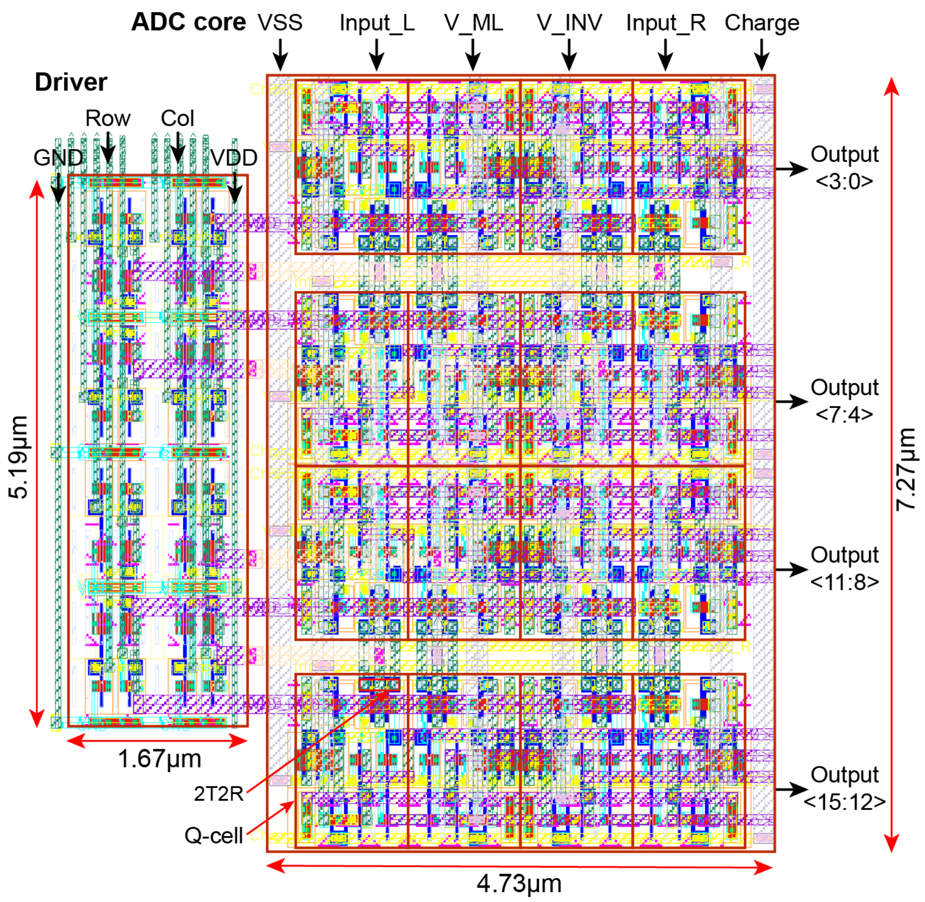

Supplementary Fig. 7. Layout of proposed 5-bit memristor-based ADC in a UMC 28 nm process. This layout, created to validate our area estimation methodology, consists of 16 Q-cells arranged in a 4×4 array and an associated program driver. The total area of the design is 43.05 μm^2, with the ADC core occupying 34.39 μm^2 and the program driver occupying 8.67 μm^2.

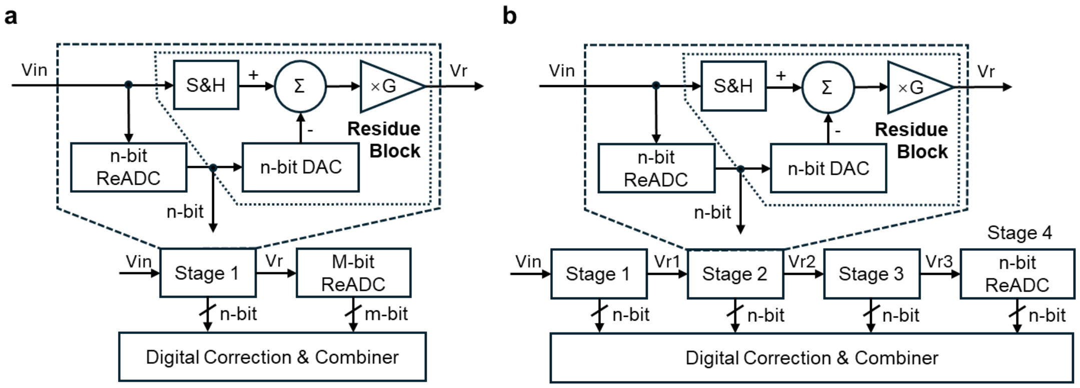

Supplementary Fig. 8. Proposed pipeline ADC architecture for high-resolution applications using proposed ADC (ReADC) as the sub-ADC. (a) Single pipeline stage architecture. The stage consists of an n-bit ReADC sub-converter, a DAC, subtraction circuit (Σ), and gain amplifier (×G). It processes input Vin to produce an n-bit digital output and passes amplified residue Vr for the next stage. **(b)** Four-stage pipeline example achieving (4×n)-bit total resolution by cascading four n-bit stages. The Digital Correction & Combiner block integrates outputs from all stages into the final high-resolution word.

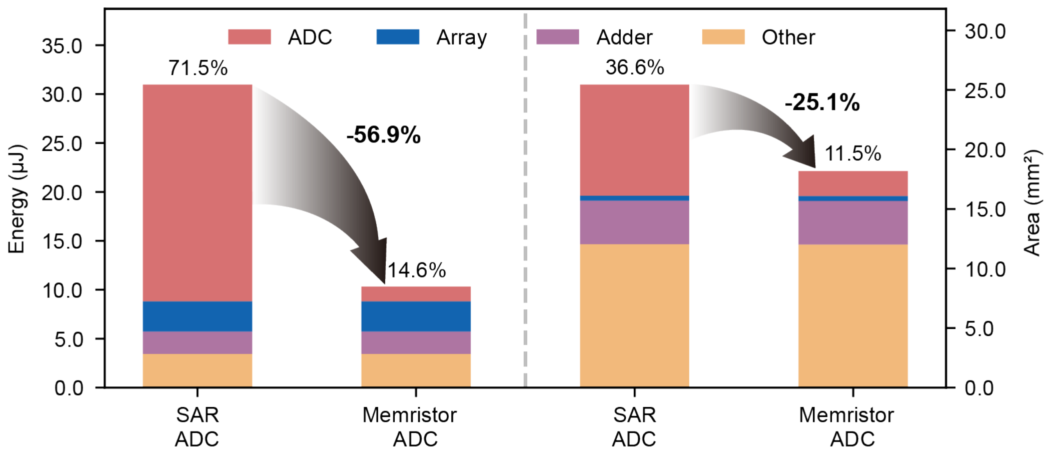

Supplementary Fig. 9. Simulated system-level energy and area analysis of ResNet18 implementation. Energy consumption and area utilization for conventional SAR ADC and proposed memristor-based ADC in CIM systems. Stacked bars illustrate the contributions of different components (ADC, Array, Adder, and Other) to total energy (left y-axis) and area (right y-axis). Arrows emphasize the percentage reduction in energy and area for the ADC portion, underscoring the advantages of the memristor-based ADC. Detailed numerical data is provided in **Supplementary Table 6**.

Q1	Q2	Q3	Q4	**Decoder Output (B2, B1, B0)**
0	1	1	1	000
0	0	1	0	001
0	0	0	1	010
0	0	0	0	011
1	0	0	0	100
1	1	0	0	101
1	1	1	0	110
1	1	1	1	111

Supplementary Table 1. Truth table for 3-bit ADC Q-cell output decoding. The decoder maps the 4-bit Q-cell outputs (Q1, Q2, Q3, Q4) to their corresponding 3-bit binary representations (B2, B1, B0).

Metric	2-bit	3-bit	4-bit	5-bit	6-bit
RMS INL (LSB)	0.142	0.117	0.159	0.319	0.717
RMS DNL (LSB)	0.164	0.118	0.210	0.419	0.940

Supplementary Table 2. Simulated memristor ADC linearity metrics (complementary to Figs. 4c-d). RMS of the maximum INL/DNL values (LSB) for 2–6 bit precisions under experimental variations. Maximum INL = 0.717 LSB (6-bit), DNL = 0.940 LSB (6-bit), all sub-1-LSB.

Supplementary Table 3. State-of-the-art ADC performance comparison for CIM applications. This table benchmarks the proposed memristor-based ADC against other ADC architectures either specifically designed for or integrated within CIM systems. Performance metrics highlight trade-offs in resolution, linearity, energy, area, and speed across different technology nodes and design approaches. N/A indicates data not available or not applicable; CAM, Content-Addressable Memory; CCO, Current-Controlled Oscillator; CVH, Current-Voltage-Hybrid; OCCS, Offset-Compensating Current Sensing; SAR, Successive Approximation Register.

Metric	This work	Yang et al. Nature Comm. 2025	Khaddam-Aljameh et al. JSSC 2022	Hsu et al. JSSC 2024	Roy et al. JSSC 2025	Yao et al. Nature 2020	Yin et al. Trans. on Electron Devices 2020	He et al. SSCL 2020	Huo et al. Nat. Electron 2022	Zhang et al. Science 2023
Type	CAM-based	Ramp-based	CCO-based	CVH-based	OCCS + SAR ADC	SAR	Flash	Flash	SAR	Ramp
Resolution (bit)	5	5	12	5	6	8	3	1	8	8
CMOS Node (nm)	16	180	14	22	22	130	90	90	55	130
ADC clk freq. (MHz)	1000	1000	3300	N/A	8.3 or 16.6	N/A	150	140	8	200
Sampling rate (MHz)	1000	31.25	N/A	N/A	N/A	N/A	18.75	17.5	0.015	0.78
Area (μm^2)	24.29	558.03	400	N/A	3180.7	1500	N/A	N/A	N/A	N/A
Power (μW)	12.58	9.3	N/A	N/A	N/A	51	N/A	N/A	33.18	11.9
Energy per Conv	12.58 fJ	42.82 pJ	N/A	N/A	N/A	10 fJ	N/A	N/A	N/A	N/A
Latency per Conv (ns)	0.66	32	N/A	N/A	N/A	10	N/A	N/A	N/A	N/A

ADC Type	Number/Macro	Area (μm²)	Energy (pJ)	Latency (ns)
Conventional SAR ADC	32	3467.38	12.00	24.00
Memristor Ramp-based ADC	1	594.48	42.82	32.00
Proposed ADC	1	24.29	1.61	21.12
	4	97.16		5.28
	8	194.32		2.64
	16	388.64		1.32
	32	777.28		0.66

Supplementary Table 4. Comparison of different ADC architectures at the macro level. Detailed performance metrics comparing the proposed simulated ADC with conventional SAR ADC model and memristor ramp-based ADC, highlighting differences in area, energy, and latency across various solutions.

CIM Component	Conventional SAR ADC		Memristor-based ADC	
	**Energy (pJ)**	**Area (μm²)**	**Energy (pJ)**	**Area (μm²)**
ADC	9736281.26	8488155.46	718105.85	1903126.12
Array	913328.27	377336.37	913328.27	377336.37
Adder	528632.61	5322160.41	528632.61	5322160.41
Other	1029142.87	3661831.26	1029142.87	3663104.57
All	12207385.02	17849483.49	3189209.60	11265727.47

Supplementary Table 5. Energy and area breakdown for VGG8 implementation. Detailed energy and area comparison data for the VGG8 network on a simulated CIM system, quantifying the improvements achieved by the proposed memristor-based ADC compared to conventional SAR ADC as visualized in **Fig. 7**.

CIM Component	Conventional SAR ADC		Memristor-based ADC	
	Energy (pJ)	Area (μm²)	Energy (pJ)	Area (μm²)
ADC	22139348.69	9320327.56	1504952.85	2089707.11
Array	3089361.76	414330.13	3089361.76	414330.13
Adder	2294402.81	3659483.27	2294402.81	3659483.27
Other	3441603.59	12051202.10	3441603.59	12020864.39
All	30964716.85	25445343.06	10330321.01	18184384.90

Supplementary Table 6. Energy and area breakdown for ResNet18 implementation. Detailed energy and area comparison data for the ResNet18 network on a simulated CIM system, quantifying the improvements achieved by the proposed memristor-based ADC compared to conventional SAR ADC as visualized in **Supplementary Fig. 9**.

Supplementary References

1. Roy, S. K. *et al.* Compute SNDR-Boosted 22-nm MRAM-Based In-Memory Computing Macro Using Statistical Error Compensation. *IEEE J. Solid-State Circuits* **60**, 1092–1102 (2025).
2. Khaddam-Aljameh, R. *et al.* HERMES-Core—A 1.59-TOPS/mm^2 PCM on 14-nm CMOS In-Memory Compute Core Using 300-ps/LSB Linearized CCO-Based ADCs. *IEEE J. Solid-State Circuits* **57**, 1027–1038 (2022).
3. Hsu, H.-H. *et al.* A Nonvolatile AI-Edge Processor With SLC–MLC Hybrid ReRAM Compute-in-Memory Macro Using Current–Voltage-Hybrid Readout Scheme. *IEEE J. Solid-State Circuits* **59**, 116–127 (2024).
